# Optimization of Cathodic Protection Design for Pre-Insulated Pipeline in District Heating System Using Computational Simulation

**DOI:** 10.3390/ma12111761

**Published:** 2019-05-30

**Authors:** Min-Sung Hong, Yoon-Sik So, Jung-Gu Kim

**Affiliations:** School of Advanced Materials Engineering, Sungkyunkwan University, 300 Chunchun-Dong, Jangan-Gu, Suwon 440-746, Korea; smith803@skku.edu (M.-S.H.); soy2871@naver.com (Y.-S.S.)

**Keywords:** cathodic protection, corrosion mitigation method, potentiodynamic polarization test, simulation, pre-insulated pipeline

## Abstract

Cathodic protection (CP) has been used as a primary method in the control of corrosion, therefore it is regarded as the most effective way for protecting buried pipelines. However, it is difficult to apply CP to a pipeline for district heating distribution systems, because the pipeline has thermally insulated coatings which could disturb the CP. Theoretical calculation and field tests alone are not enough for a reliable CP design, and therefore additional CP design methods such as computational analysis should be used. In this study, the CP design for pre-insulated pipelines is tested considering several environmental factors, such as temperature and coating defect ratio. Additionally, computational analysis is performed to verify and optimize the CP design. The simulation results based on theoretical methods alone failed to satisfy the CP criteria. Then, a re-design is conducted considering the IR drop. Consequently, all of the simulation results of defective pipelines satisfied the CP criteria after adding the proper CP current.

## 1. Introduction

**Figure 1:**
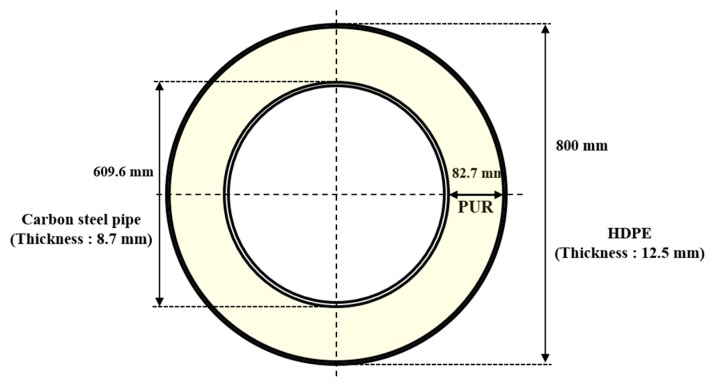
Schematic diagram of the pre-insulated coated pipe (600 A).

In district heating (DH) systems, heated water is distributed through a double-pipe network and transferred to buildings for use in space heating, hot water generation, and process heating [1]. DH systems have three main elements: the heat source, the distribution system, and the customer interface. The distribution system supplies hot water from the heat source to the heat consumer and returns with temperatures in the range of 40 °C to 120 °C [2]. Generally, pipelines in DH distribution systems use a thermally insulated coating to minimize heat loss during transfer. As shown in Figure 1, the coating consists of two layers: an inner layer of polyurethane foam (PUR) to reduce heat loss, and an outer layer of high-density polyethylene (HDPE) to protect the PUR [3]. The coatings effectively mitigate corrosion by blocking the outer environment, which contains corrosive elements such as water, oxygen, and chloride ions, when the coating is maintained perfectly. However, the HDPE is susceptible to unpredictable mechanical damage, and the PUR can be vanished by heat, humidity, and oxygen during its long operational life [4,5]. Several studies have reported that the main source of corrosion is groundwater introduced through failure of the HDPE and PUR [5,6,7]. 

Cathodic protection (CP) has been used as the primary method in the control of corrosion, in conjunction with protective coatings. CP can reduce the corrosion rate, and a properly maintained system will provide protection in accordance with the designed structural life [8]. The impressed current CP (ICCP) system has a power supply (rectifier) that generates larger potential differences between the anode and the structure [9]. For this reason, ICCP is applied to many industrial pipelines. However, despite the availability of CP, there are still several limitations in applying CP to pre-insulated pipelines [10]. The National Association of Corrosion Engineers (NACE) reported that the CP design for pre-insulated pipelines is ineffective because the protection current cannot reach the corroded area through the insulating layer [11]. Additionally, according to previous studies, the corrosion rate of PUR-insulated carbon steel is much lower than that of uninsulated (bare) steel, even when the PUR is fully immersed in groundwater [12]. Therefore, CP for pre-insulated pipelines may be unnecessary when the PUR layer is intact. However, the immersed PUR layer can deteriorate and vanish during long operating periods, causing exposure of the bare carbon steel to the corrosive environment. For this reason, it is important to apply CP to operating pipelines with external coating defects, as a precaution against sudden fracture. Nevertheless, it is difficult to design CP systems for operating pipelines using only theoretical methods and a limited number of standards. It is also difficult to verify the appropriate protecting current required to reach the external surface of the pipeline with proper CP potential. For this reason, additional CP design methods, such as computational analysis, should be applied to optimize the design [13,14,15]. To improve the reliability of simulation results, several essential factors should be considered, such as polarization data for real materials and appropriate environmental information.

In this study, a CP system was designed for an existing pipeline with damaged insulation, taking into consideration environmental factors, such as corrosion properties of real materials, operating temperatures, and structural effects. Additionally, electrochemical tests were performed in synthetic groundwater to obtain input data for the computer simulation. Finally, a computational analysis was performed to verify and optimize the CP design of pre-insulated pipelines.

## 2. Materials and Methods 

### 2.1. Materials and Test Conditions

The corrosion environment used was synthetic groundwater. Table 1 gives the chemical composition of the synthetic groundwater, and HNO_3_ was used to control the pH of the solution. A welded carbon steel specimen consisting of a base metal, heat affected zone, and a weld metal was used during testing to calculate the required CP current. Table 2 shows the chemical composition of the SPW400 (carbon steel), and Table 3 shows the welding methods used in all experiments. The surface of the specimen was polished with 600-grit silicon carbide (SiC) paper, degreased with ethanol, and dried with N_2_.

### 2.2. Electrochemical Test Methods

All electrochemical experiments were performed using a three-electrode system, in a 1000 mL Pyrex glass corrosion cell connected to an electrochemical apparatus. The test specimens were connected to a working electrode, a graphite rod was used as the counter electrode, and a saturated calomel electrode (SCE) was used as the reference electrode. The area of the test specimen exposed to the electrolyte was 2.25 cm^2^ (1.5 cm × 1.5 cm). An open-circuit potential (OCP) was established within three hours to carry out the electrochemical test. Potentiodynamic polarization tests were carried out in accordance with ASTM G5-14 (Standard Reference Test Method for Making Potentiodynamic Anodic Polarization Measurements), using a VMP2 (Bio-Logic Science Instruments, Seyssinet-Pariset, France) with a potential sweep of 0.166 mV/sec, from an initial potential of −2000 mV versus the reference to a final potential of 200 mV versus the OCP. The electrochemical tests were performed at 80 °C, because a previous study found that the highest protection current for carbon steel was required at this temperature [7].

### 2.3. CP Design and Computational Analysis Method

The computational analysis tool BEASY S/W (BEASY Ltd., Southampton, England), which is based on the boundary element method (BEM), was used to conduct 3D modeling and computational analysis of the pre-insulated pipeline. The required CP current (I_req_) for the pipeline was calculated, taking into consideration the current density of real material measured by electrochemical tests. The cathodic polarization curve, which was used as input data for the simulation, was obtained from the potentiodynamic polarization test, which incorporated the environmental information.

## 3. Results and Discussion

### 3.1. Potentiodynamic Polarization Tests

**Figure 2:**
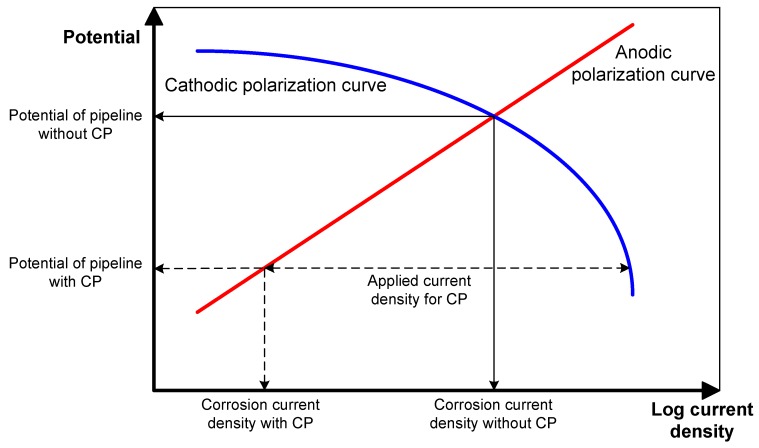
Evan’s diagram, indicating the relationship between the applied current density and protection potential [16].

**Figure 3:**
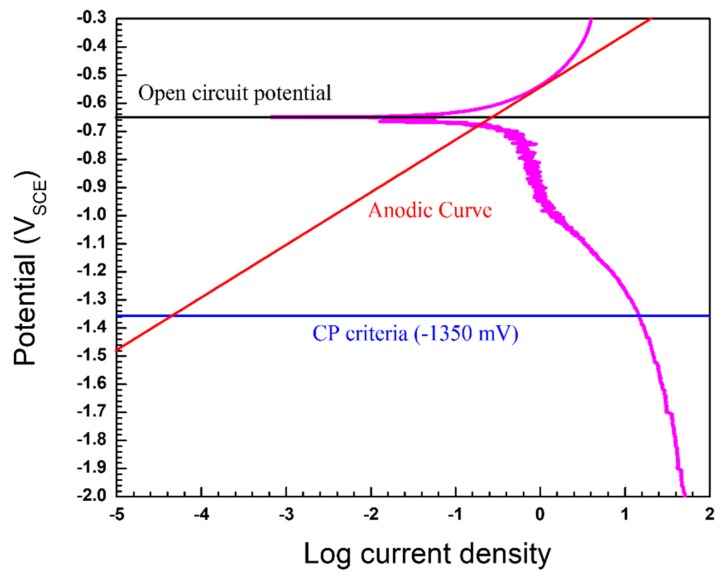
Potentiodynamic polarization curves in the synthetic groundwater at 80 °C.

The applied current density (i_app_) for the pre-insulated pipeline was calculated using the Evans diagram, as shown in Figure 2. According to the diagram, anodic current density is under activation control (activation polarization), and cathodic current density is limited at a higher current density (concentration polarization). As the applied current density for CP is increased, the potential and the corrosion current density are reduced simultaneously [16,17]. According to the previous study, since the pre-insulated pipeline has a high corrosion rate at 80 °C, the reasonable maximum CP potential is −1350 mV_SCE_ [7]. Figure 3 shows the results of the potentiodynamic polarization test in synthetic groundwater at 80 °C. The corrosion current density was determined using the Tafel extrapolation method. Table 4 shows the calculated CP current density, which will apply to the CP design. The applied current density was calculated as the difference between the anodic polarization curve and cathodic polarization curve at −1350 mV_SCE_, as shown in Figure 2. The cathodic polarization curve, which contains the corrosion properties of real material, was used as the input data for computational analysis.

### 3.2. Cathodic Protection Design and Computational Analysis

**Figure 4:**
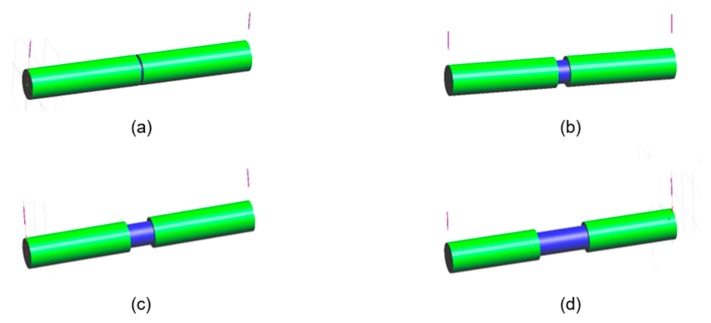
3D modeling of pipeline according to the defect ratio: (**a**) 1%, (**b**) 5%, (**c**) 10%, (**d**) 20%.

The pre-insulated pipeline was connected every 6 m by welds, therefore, the CP design was preformed to 6 meters of 600 A pipe (Figure 1). In addition, ICCP anodes were installed at both edges of the pipeline, which are the parts most sensitive to corrosion because it will connect using welding. In this study, the CP design was applied to operating pipelines with slight defects. Therefore, the CP design was tested at a range of defect ratios (1, 5, 10, 20%), and it is assumed that the insulating part of the pipeline has no defect. Figure 4 shows the 3D modeling of the pipeline according to defect ratio. For modeling and calculations, the approach was based on the assumption that the crevice between the coating and pipeline was not effective as a CP [18]. Table 5 shows the basic design parameters related to the structural factors. The surface area of the pipe used in the CP design was 12.62 m^2^, which included an additional 10% safety factor. The resistivity of soil was assumed to be 1000 Ω∙cm, corresponding to a highly corrosive environment. The required current (I_req_) for CP was calculated from the following equation [19,20]:

I_req_ = C_defect_ · i_app_ · A_pipe,_ (1)

where C_defect_ is the defect ratio of the pipeline, i_app_ is the applied current density of the pipe material calculated from the electrochemical test, A_pipe_ is the surface area of the pipe. I_req_ is calculated with the defect ratio, as listed in Table 6.

**Figure 5:**
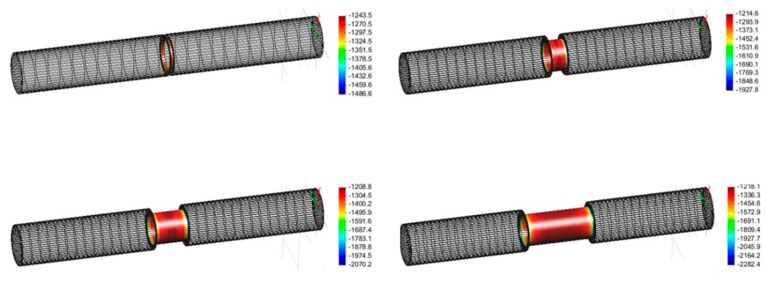
Simulation results (averaged protection potential, mV_SCE_) according to the defect ratio: (**a**) 1%, (**b**) 5%, (**c**) 10%, (**d**) 20%.

The computational analysis was performed using the cathodic polarization curve data, obtained from the electrochemical tests. Figure 5 shows the simulation results for CP. All of the simulation results failed to satisfy the CP criteria for pre-insulated pipelines (under −1350 mV_SCE_) because the IR drop caused by soil and structural factors was not considered in the CP design.

**Figure 6:**
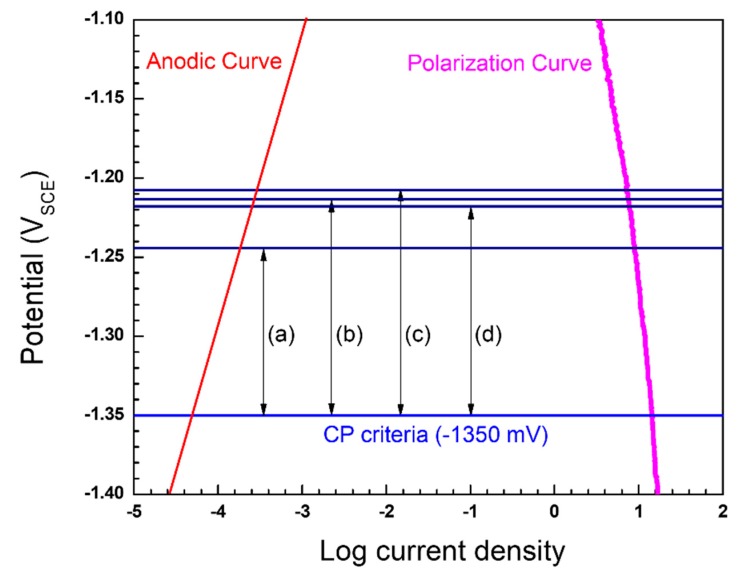
Calculation of additional current caused by IR drop in the polarization curve: potential difference of (**a**) 1%, (**b**) 5%, (**c**) 10%, (**d**) 20% defected pipelines.

The additional CP current required to satisfy the CP criteria should be calculated taking into consideration the polarization curve, as shown in Figure 6. The maximum CP potentials were defined based on the simulation results according to the defect ratio. Then, the applied current densities were calculated at the maximum CP potential from the simulation results, using the same method as above. To obtain the additional CP current densities, the difference was calculated between the calculated applied current densities, according to the defect ratio and applied current density at −1350 mV_SCE_. The additional CP currents were then calculated using Equation (1). The calculated values are listed in Table 7. 

**Figure 7:**
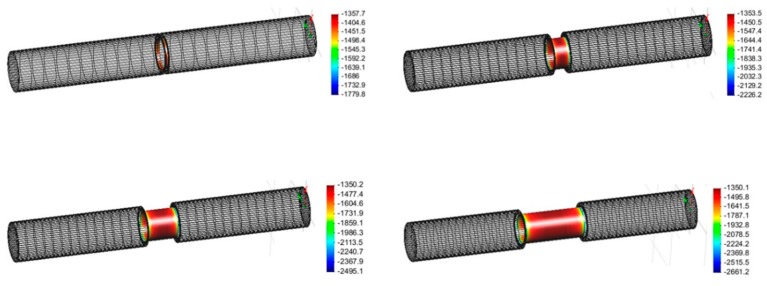
Optimized simulation results (averaged protection potential, mV_SCE_) according to the defect ratio: (**a**) 1%, (**b**) 5%, (**c**) 10%, (**d**) 20%.

Then, the entire simulation was re-conducted. Figure 7 shows the optimized simulation results, and it was verified that all of the pipelines with different defect ratios satisfied the CP criteria. Another important point is over-protection due to the low CP criteria of district pipelines. The simulation results show that the minimum CP potentials have a range from −1.7 V_SCE_ to −2.6 V_SCE_. This is quite a low potential value, which could cause hydrogen embrittlement risk. However, according to the international standards, such as NACE (RP0169-96), ARAMCO (SAES-X-400), and BSI (BS 7361-1), the over protection range of the steel pipeline ranges from −2.5 V_SCE_ to −5 V_SCE_. Therefore, the simulation results can apply up to 10% of the defect ratio, which has a minimum potential of about −2.49 V_SCE_. When the CP applies over 10% of the defect ratio, the site of defect should be previously investigated. Then, the anode should be installed as close as possible to the defect area, to avoid over protection and reduce CP current requirement. Therefore, the investigation of the defect area is one of the significant design parameters in practical CP installation.

## 4. Conclusions

In this study, a credible CP design method for existing pre-insulated pipelines was conducted, taking into consideration the environmental factors, and computational analysis was performed to verify and optimize the CP design. According to the results, the following conclusions were drawn:

♦ The results of the simulations using the theoretical method failed to satisfy the CP criterion determined for heating pre-insulated pipeline. To solve the problem, a re-design was conducted, taking into consideration the IR drop caused by soil and structural factors. Consequently, after adding the proper CP current, all of the simulation results of defective pipelines satisfied the CP criteria.
♦ Incorporating practical corrosion properties of metal and environmental factors in the computational analysis improves the reliability of the CP design for a pipeline. For this reason, application of CP is recommended for pre-insulated pipelines, to mitigate external corrosion and reduce maintenance costs. The computational analysis is an essential step for credible CP design.

## Figures and Tables

**Table 1:** Chemical composition of synthetic groundwater.

CaCl_2_ (ppm)	MgSO_4_∙7H_2_O (ppm)	NaHCO_3_ (ppm)	H_2_SO_4_ (ppm)	HNO_3_ (ppm)	pH	Resistivity (kΩ∙cm)
133.2	59	208	85	22.2	6.8	1.736

**Table 2:** Chemical composition of SPW400 (wt.%).

Fe	C	P	S
Balance	0.25 Max.	0.04 Max.	0.04 Max.

**Table 3:** Welding procedure specification.

Welding Process	GTAW
Joint design	Single V joint with a 60° included angle and a 1.6 mm root face
Electrode	GTAW ER70S-G
Voltage	12–15 V
Current	100–180 A
Polarity	Direct Current Straight Polarity (DCSP)
Travel speed	20–30 cm/min
Welding atmosphere	Ar, 15–25 L/min

**Table 4:** Results of Potentiodynamic Polarization Test at 80 °C in the Synthetic Groundwater.

Corrosion Potential (E_corr_, mV_SCE_)	Corrosion Current Density (i_corr_, A/m^2^)	Βc (mV)	Βa (mV)	Applied Current Density (i_app_, A/m^2^)
−649	0.493	258.3	78.2	14.45

**Table 5:** Basic design parameters related to the structural factors.

**Pipeline** **(600 A)**	**Diameter**	609.6 mm
	**Length**	6 m
	**Surface Area**	11.48 m^2^
**Resistivity of Soil**		1000 Ω∙cm
**Temperature on the Pipeline**		80 °C
**CP Criteria**		Under −1350 mV

**Table 6:** Required current calculation for cathodic protection (CP).

Applied Current Density (i_app_)	Surface Area with 10% Safety Factor (A_pipe_)	Defect Ratio (C_defect_)		Required Current (I_req_)
14.45 A/m^2^	12.62 m^2^	1%	0.01	1.824 A
		5%	0.05	9.120 A
		10%	0.1	18.241 A
		20%	0.2	36.483 A

**Table 7:** Results of calculated additional CP current and optimized current for CP.

Defect Ratio	Max. Potential in Previous Results	Additional Current Density	Additional Current	Optimized Current for CP
1%	−1243.5 mV	7.079 A/m^2^	0.893 A	2.717 A
5%	−1214.6 mV	12.589 A/m^2^	7.944 A	17.064 A
10%	−1208.8 mV	13.804 A/m^2^	17.420 A	35.661 A
20%	−1218.1 mV	12.303 A/m^2^	31.052 A	67.535 A
